# Expanding the clinical spectrum of biglycan-related Meester-Loeys syndrome

**DOI:** 10.1038/s41525-024-00413-z

**Published:** 2024-03-26

**Authors:** Josephina A. N. Meester, Anne Hebert, Maaike Bastiaansen, Laura Rabaut, Jarl Bastianen, Nele Boeckx, Kathryn Ashcroft, Paldeep S. Atwal, Antoine Benichou, Clarisse Billon, Jan D. Blankensteijn, Paul Brennan, Stephanie A. Bucks, Ian M. Campbell, Solène Conrad, Stephanie L. Curtis, Majed Dasouki, Carolyn L. Dent, James Eden, Himanshu Goel, Verity Hartill, Arjan C. Houweling, Bertrand Isidor, Nicola Jackson, Pieter Koopman, Anita Korpioja, Minna Kraatari-Tiri, Liina Kuulavainen, Kelvin Lee, Karen J. Low, Alan C. Lu, Morgan L. McManus, Stephen P. Oakley, James Oliver, Nicole M. Organ, Eline Overwater, Nicole Revencu, Alison H. Trainer, Bhavya Trivedi, Claire L. S. Turner, Rebecca Whittington, Andreas Zankl, Dominica Zentner, Lut Van Laer, Aline Verstraeten, Bart L. Loeys

**Affiliations:** 1 Center of Medical Genetics, University of Antwerp and Antwerp University Hospital, Antwerp, Belgium; 2 Department of Clinical Genetics, Chapel Allerton Hospital, Leeds Teaching Hospitals, NHS Foundation Trust, Leeds, UK; 3 Genomic and Personalized Medicine, Atwal Clinic, Palm Beach, FL USA; 4 Department of Internal and Vascular Medicine, CHU Nantes, Nantes Université, Nantes, France; 5 Service de Médecine Génomique des Maladies Rares, Groupe Hospitalier Universitaire Centre, Paris, Assistance Publique Hôpitaux de Paris, Paris, France; 6 Université de Paris Cité, Inserm, PARCC, Paris, France; 7 Department of Vascular Surgery, Amsterdam University Medical Center, Amsterdam, The Netherlands; 8 Northern Genetics Service, Newcastle upon Tyne Hospitals NHS Foundation Trust, Newcastle upon Tyne, UK; 9 GeneDx LLC, Gaithersburg, MD USA; 10 Division of Human Genetics, Children’s Hospital of Philadelphia, Philadelphia, PA USA; 11 Service de Génétique Médicale, CHU Nantes, Nantes, France; 12 Bristol Heart Institute, University Hospitals Bristol & Weston NHS Foundation Trust, Bristol, UK; 13 Department of Medical Genetics & Genomics, AdventHealth Medical Group, Orlando, FL USA; 14 South West Genomic Laboratory Hub, Bristol Genetics Laboratory, Bristol, UK; 15 North West Genomic Laboratory Hub, Manchester Centre for Genomic Medicine, Manchester, UK; 16 Hunter Genetics, Waratah, NSW Australia; 17 Leeds Institute of Medical Research, University of Leeds, Leeds, UK; 18 Department of Human Genetics, Amsterdam University Medical Center, Vrije Universiteit Amsterdam, Amsterdam, The Netherlands; 19 Clinical Genetics Service, University Hospitals Bristol and Weston NHS Foundation Trust, Bristol, UK; 20 Department of Cardiology, Heart Centre Hasselt, Jessa Hospital, Hasselt, Belgium; 21 Department of Clinical Genetics, Research Unit of Clinical Medicine, Medical Research Center Oulu, Oulu University Hospital and University of Oulu, Oulu, Finland; 22 Department of Medical and Clinical Genetics, University of Helsinki and Helsinki University Hospital, Helsinki, Finland; 23 Clinical Genetics Department, University Hospitals Bristol and Weston NHS Foundation Trust St Michael’s Hospital, Bristol, UK; 24 University of Bristol, Canynge Hall, Bristol, UK; 25 John Hunter Hospital, New Lambton Heights, NSW Australia; 26 College of Health, Medicine and Wellbeing, School of Medicine, University of Newcastle, Newcastle, NSW Australia; 27 Genomic Diagnostics Laboratory, Manchester Centre for Genomic Medicine, Manchester, UK; 28 Department of Genetics, University Medical Center Groningen, Groningen, The Netherlands; 29 Center for Human Genetics, Cliniques Universitaires Saint-Luc and Université Catholique de Louvain, Brussels, Belgium; 30 Department of Genomic Medicine, The Royal Melbourne Hospital and University of Melbourne, Parkville, Melbourne, VIC Australia; 31 Department of Clinical Genetics, Royal Devon and Exeter NHS Foundation Trust, Exeter, UK; 32 Children’s Hospital at Westmead Clinical School, Faculty of Medicine and Health, University of Sydney, Sydney, NSW Australia; 33 Department of Clinical Genetics, Children’s Hospital at Westmead, Sydney, NSW Australia; 34 Garvan Institute of Medical Research, Sydney, NSW Australia; 35 Department of Clinical Genetics, Radboud University Medical Center, Nijmegen, The Netherlands

**Keywords:** Aneurysm, Disease genetics, Medical genomics, Mutation

## Abstract

Pathogenic loss-of-function variants in *BGN*, an X-linked gene encoding biglycan, are associated with Meester-Loeys syndrome (MRLS), a thoracic aortic aneurysm/dissection syndrome. Since the initial publication of five probands in 2017, we have considerably expanded our MRLS cohort to a total of 18 probands (16 males and 2 females). Segregation analyses identified 36 additional *BGN* variant-harboring family members (9 males and 27 females). The identified *BGN* variants were shown to lead to loss-of-function by cDNA and Western Blot analyses of skin fibroblasts or were strongly predicted to lead to loss-of-function based on the nature of the variant. No (likely) pathogenic missense variants without additional (predicted) splice effects were identified. Interestingly, a male proband with a deletion spanning the coding sequence of *BGN* and the 5’ untranslated region of the downstream gene (*ATP2B3*) presented with a more severe skeletal phenotype. This may possibly be explained by expressional activation of the downstream ATPase *ATP2B3* (normally repressed in skin fibroblasts) driven by the remnant *BGN* promotor. This study highlights that aneurysms and dissections in MRLS extend beyond the thoracic aorta, affecting the entire arterial tree, and cardiovascular symptoms may coincide with non-specific connective tissue features. Furthermore, the clinical presentation is more severe and penetrant in males compared to females. Extensive analysis at RNA, cDNA, and/or protein level is recommended to prove a loss-of-function effect before determining the pathogenicity of identified *BGN* missense and non-canonical splice variants. In conclusion, distinct mechanisms may underlie the wide phenotypic spectrum of MRLS patients carrying loss-of-function variants in *BGN*.

## Introduction

Meester-Loeys syndrome (MRLS, MIM #300989) is an X-linked thoracic aortic aneurysm and dissection (TAAD) syndrome caused by loss-of-function variants in the biglycan gene (*BGN*). This syndrome was first described in five families in 2017^1^. Early-onset aortic aneurysm and dissection of the aortic root or more distal ascending aorta were reported as the main clinical features. Beyond the aorta, aneurysms in the brain, pulmonary artery, and ductus arteriosus were described. Considerable clinical overlap with Marfan syndrome (MFS, MIM #154700)^2,3^ and Loeys-Dietz syndrome (LDS, MIM #609192, #610168, #613795, #614816, #615582, #619656)^4–11^ was noted. LDS-overlapping features were hypertelorism, bifid uvula, and cervical spine instability. Other recurrent connective tissue features included pectus deformities, joint hypermobility, and contractures, as well as striae. Unique MRLS features, not typically seen in MFS or LDS, included ventriculomegaly, relative macrocephaly, hypertrichosis, and gingival hypertrophy. Mild skeletal dysplasia, characterized by hip dislocation, platyspondyly, phalangeal dysplasia, and dysplastic epiphyses of the long bones, was also reported in males with a deletion of the coding part of *BGN*. Due to the X-linked nature of the disorder, the phenotype in females varied greatly, ranging from unaffected upon repeated echocardiographic evaluation to death due to aortic dissection^12^. Strikingly, biglycan deficiency in BALB/cA mice was reported to lead to sudden death due to spontaneous aortic dissection/rupture in 50% of the males by twelve weeks of age^13^.

Biglycan is a small leucine-rich class I proteoglycan that is involved in the maintenance and assembly of the extracellular matrix (ECM)^14^. The small protein core contains ten leucine-rich repeats, to which two tissue-specific chondroitin or dermatan-sulfate glycosaminoglycan (GAG) chains are attached^15^. Through these GAG chains and its core region, biglycan interacts with other ECM proteins, including collagen type I, II, III, and VI as well as elastin^16–18^. Aside from mechanically linking matrix components, biglycan is also involved in the regulation of growth factor signaling such as bone morphogenetic proteins and transforming growth factor beta^19^. Biglycan is expressed in various tissues (e.g., bone, skin, heart, lung, artery) and specialized cell types (e.g., endothelial cells, skeletal myocytes, differentiating keratinocytes)^20,21^. Importantly, it is one of the most highly expressed genes in the human aorta (www.gtexportal.org).

Over the past few years, we have considerably expanded our *BGN*-related MRLS patient cohort. Here, we report hemizygous and heterozygous *BGN* variants associated with MRLS, an in-depth molecular investigation, extended clinical phenotype descriptions, and genotype-phenotype associations.

## Results

### Identification and investigation of candidate *BGN* variants

**Fig. 1:**
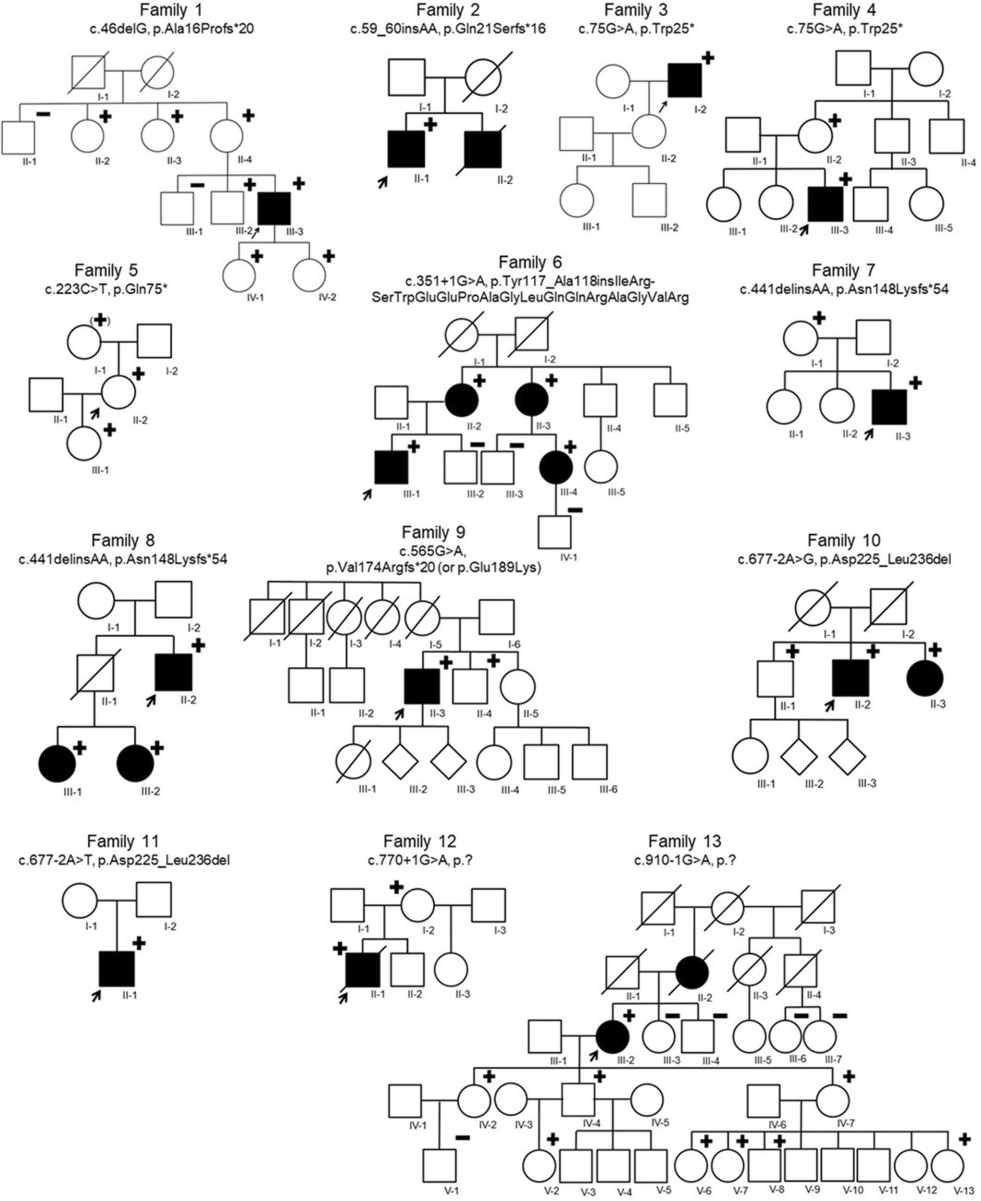
Pedigrees of thirteen families with *BGN* variants identified in the current study. Circle = female. Square = male. Diamond = unknown sex. Filled = patients with connective tissue features. Unfilled = unaffected or asymptomatic person. Strikethrough = deceased person. + = hemi-/heterozygous *BGN* variant carrier. (+) = heterozygous *BGN* variant carrier reported by proband. – = tested negative for *BGN* variant. Arrow = proband.

Since the initial publication of five families with pathogenic variants in *BGN* in 2017^1^, thirteen additional families with *BGN* variants were identified (Fig. 1 and Table 1).

**Table 1:** Meester-Loeys syndrome cohort extension led to the identification of *BGN* variants in thirteen additional families

Family ID	cDNA change	Protein change	Variant effect	Variant classification	Classification criteria
1	c.46delG	p.Ala16Profs*20	Frameshift	Likely pathogenic variant	pathogenic very strong (PVS) 1, pathogenic moderate (PM) 2
2	c.59_60insAA	p.Gln21Serfs*16	Frameshift	Likely pathogenic variant	PVS1, PM2
3	c.75 G > A	p.Trp25*	Nonsense	Likely pathogenic variant	PVS1, PM2
4	c.75 G > A	p.Trp25*	Nonsense	Likely pathogenic variant	PVS1, PM2
5	c.223 C > T	p.Gln75*	Nonsense	Likely pathogenic variant	PVS1, PM2
6	c.351+1 G > A	p.Tyr117_Ala118insIleArgSerTrpGluGluProAlaGly-LeuGlnGlnArgAlaGlyValArg	Splice site, in-frame insertion	Variant of unknown significance	PM2, PM4
7	c.441delinsAA	p.Asn148Lysfs*54	Frameshift	Variant of unknown significance	PVS1
8	c.441delinsAA	p.Asn148Lysfs*54	Frameshift	Variant of unknown significance	PVS1
9	c.565 G > A	p.Val174Argfs*20 or p.Glu189Lys	Missense, splice site, frameshift	Likely pathogenic variant	PVS1_strong, PM2, PP3
10	c.677-2 A > G	p.Asp225_Leu236del	Splice site, in-frame deletion	Likely pathogenic variant	PM2, PM4_strong, pathogenic supporting (PP) 1
11	c.677-2 A > T	p.Asp225_Leu236del	Splice site, in-frame deletion	Likely pathogenic variant	PM2, PM4_strong
12	c.770+1 G > A	p.?	Splice site, predicted frameshift	Likely pathogenic variant	PVS1_strong, PM2
13	c.910-1 G > A	p.?	Splice site, predicted frameshift	Likely pathogenic variant	PVS1_strong, PM2

#### Frameshift

**Fig. 2:**
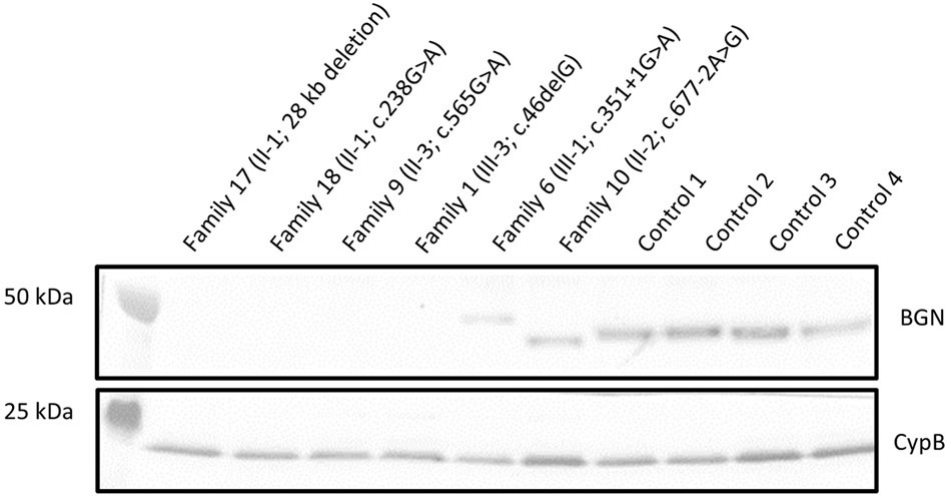
Western Blot of BGN protein expression in skin fibroblasts of probands from families 1, 6, 9, 10, 17, and 18 as well as matched controls. Intracellular proteins were isolated from skin fibroblast samples and the biglycan (BGN) protein content was visualized. Cyclophilin B (CypB) was used as a loading control. Family 17 and 18 were reported as family 4 and 5, respectively, by Meester et al.^1^. Controls 1–4 were samples of age-matched male controls. The Western Blot was derived from one experiment and all lanes were processed in parallel.

In families 1 and 2, likely pathogenic frameshift variants were identified (family 1: c.46delG, p.Ala16Profs*20, SCV004170959; family 2: c.59_60insAA, p.Gln21Serfs*16, SCV004170960). Skin fibroblasts were available from the proband of family 1 (1-III-3). Although we could not confirm the occurrence of nonsense-mediated mRNA decay (NMD), we did not observe any biglycan protein expression by western blot (Fig. 2). Another identical frameshift variant was identified in families 7 and 8 (c.441delinsAA, p.Asn148Lysfs*54, SCV004170964). This frameshift variant is present as two separate entries (rs782449715 and rs782199865) in GnomAD in three individuals^22^. Intriguingly, these three GnomAD individuals (one female and two males) are all part of the European Finnish population, and both probands of families 7 and 8 originate from (Northern) Finland. Western Blot on protein derived from skin fibroblasts of the male proband of family 7 (7-II-3) confirmed the predicted complete absence of the biglycan protein (Supplementary Fig. 1). Although a small fraction of mutant peaks was observed with cDNA analysis of the variant-harboring unaffected mother (7-I-1), no mutant or decreased concentration of biglycan protein was observed by Western Blot (Supplementary Fig. 1). No skin fibroblasts from family 8 were available for further testing of this *BGN* frameshift variant.

#### Nonsense

In the probands of families 3 to 5, likely pathogenic nonsense variants were identified (families 3 and 4: c.75 G > A, p.Trp25*, SCV004170961; family 5: c.223 C > T, p.Gln75*, SCV004170962), which are predicted to lead to NMD and biglycan protein absence. No skin fibroblasts of the probands or family members were available for further testing of this hypothesis.

#### Missense

In family 9, a missense variant was identified (c.565 G > A, p.Glu189Lys, SCV004170965). Since this variant affects the last nucleotide of exon 4, and thus a canonical splice site, cDNA analysis of RNA derived from skin fibroblasts of the male proband (9-II-3) was performed to determine the effect of this variant. We observed an alternatively spliced transcript that lacks the last 46 nucleotides of exon 4 due to the use of an exonic, cryptic splice site, resulting in a frameshift effect (p.Val174Argfs*20) and that partly undergoes NMD. Furthermore, the wildtype spliced transcript (containing the missense variant) was also detected on the cDNA level. Nonetheless, no biglycan protein expression was observed by western blot (Fig. 2), confirming that this variant causes a complete loss of the biglycan protein.

#### Splice site

In families 6 and 10 to 13, splice site variants were identified. The variant of family 6 affects the first nucleotide of intron 3 (c.351+1 G > A, SCV004170963). cDNA analysis of RNA derived from skin fibroblasts of the male proband (6-III-1) showed the use of a cryptic splice site in intron 3, leading to an in-frame insertion of 51 nucleotides and, consequently, an addition of 17 amino acids (p.Tyr117_Ala118insIleArgSerTrpGluGluProAlaGlyLeuGlnGlnArgAlaGlyValArg). The presence of this aberrant (longer) protein was also observed by Western Blot (Fig. 2), indicating that this mutant biglycan protein is not (completely) degraded. No normal biglycan protein was observed. In families 10 and 11, a splice site variant affecting the same nucleotide, but resulting in a different substitution, was identified (family 10: c.677-2 A > G, SCV004170966; family 11: c.677-2 A > T, SCV004170967). Skin fibroblasts of the male probands from these two families (10-II-2 and 11-II-1) were available for cDNA and Western Blot analysis. The *BGN* variants identified in families 10 and 11 were shown to result in the activation of a cryptic splice site in exon 6, leading to the loss of the first 33 nucleotides of that exon, and, consequently, the in-frame loss of 11 amino acids (p.Asp225_Leu236del). This aberrant shorter biglycan protein was confirmed by Western Blot (Fig. 2 and Supplementary Fig. 1). No normal biglycan protein was observed. The variants identified in families 12 and 13 affect the canonical donor splice site of exon 6 and the acceptor splice site of exon 8, respectively (family 12: c.770+1 G > A, SCV004170968; family 13: c.910-1 G > A, SCV004170969). Although no skin fibroblasts were available for further testing, splice prediction algorithms predict distinct effects on splicing. For family 12, the donor splice site is predicted to be lost, potentially leading to the skipping of exon 6, which would result in a frameshift. For family 13, a novel splice site is predicted to be located one nucleotide further downstream than the canonical splice site, also leading to a frameshift. Since exon 8 is the last exon of Biglycan, no NMD is predicted to occur. Yet, the N-terminal protein structure would be altered because of the variant.

### Further investigation of previously identified *BGN* variants

The following two families (17 and 18) were already reported by Meester et al.^1^, but in this study, we performed additional assays to determine the effect of the different reported *BGN* variants on protein level by Western Blot. The male proband of family 17 (family 4 in Meester et al.^1^) carries a 28 kb deletion of the coding part of biglycan (ChrX(GRCh38):g.153502980_153530518del; VCV000265797.1). Western Blot experiments confirmed the absence of the biglycan protein in this patient (Fig. 2). The splice site variant in family 18 (family 5 in Meester et al.^1^; c.238 G > A, p.Gly80Ser; VCV000265798.2) was described to result in four different splice products, of which two underwent NMD. The other two, less abundant, splice products did not lead to NMD. However, no biglycan protein expression was observed by western blot (Fig. 2), indicating a complete loss of the biglycan protein in this male patient (18-II-1).

#### BGN 5’-UTR hijacking by ATP2B3

**Fig. 3:**
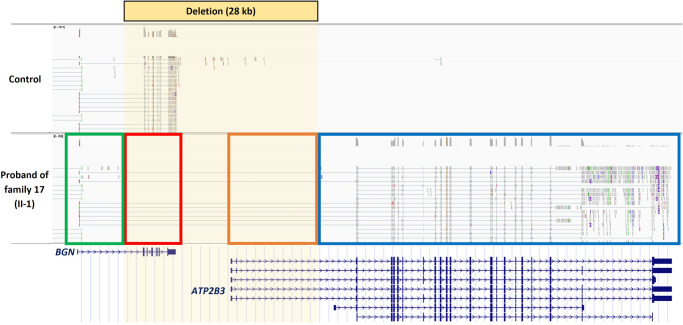
mRNA sequencing of skin fibroblasts of the proband from family 17 (17-II-1) shows *BGN* 5’-UTR hijacking by *ATP2B3.* Family 17 was reported as family 4 by Meester et al. (2017)^1^. The control sample was age- and sex-matched.

The 28 kb deletion in the proband of family 17 (17-II-1; family 4 in Meester et al.^1^) abolishes the coding part of *BGN* (exon 2-8). In addition, the deletion affects a portion of DNA downstream of *BGN*, containing the start of the 5’ untranslated region (5’-UTR) of several *ATP2B3* isoforms. RNA sequencing on the skin fibroblasts of this patient was performed to investigate the effect of this deletion in more detail. As expected, no expression of the coding exons of *BGN* was observed (Fig. 3, red box). However, the 5’-UTR of *BGN* remained expressed (Fig. 3, green box). Interestingly, due to the partial loss of its own 5’-UTR (Fig. 3, orange box), *ATP2B3* now uses the 5’-UTR (exon 1; Fig. 3, green box) of *BGN* to drive its expression (Fig. 3, blue box). *ATP2B3* is not expressed in healthy control skin fibroblasts, but due to hijacking of the 5’-UTR of *BGN*, it is now expressed in this cell type as a consequence of the 28 kb deletion in this patient.

### Cohort characteristics

Combining the previously published cohort^1^ with the additional thirteen families, our cohort now includes 18 probands (16 males and 2 females) with an average age at presentation of 33 years, ranging from 0 to 70 years. Thirteen male probands presented with aortic (*n* = 10) and/or widespread arterial (*n* = 8) aneurysms/dissections, a male newborn (4-III-3, 0.5 years) presented with marked hydrocephaly, craniofacial features, pectus excavatum and syndactyly, a tall male (6-III-1, 36 years) with significant camptodactyly, spine deformities, flat feet, and joint contractures, and another male (7-II-3, 13 years) presented with various syndromic findings but without cardiovascular features (yet). Our cohort comprises two female probands: one (13-III-2, 59 years) presented with an aortic aneurysm, whereas the other (5-II-2, 44 years) was detected as part of a comprehensive prenatal testing study. Segregation analyses identified 36 additional *BGN* variant-harboring family members (9 males and 27 females). The clinical phenotype of female *BGN* variant carriers ranged from no phenotype to aortic aneurysm with typical MRLS connective tissue features. A summary of the clinical features of all *BGN* variant-harboring individuals can be found in Table 2. A detailed overview of the clinical features of the individuals from families 1 to 13 can be found in Supplementary Table 1 and the Supplementary Notes containing the family case reports, and for families 14 to 18 in Meester et al.^1^.

**Table 2:** Clinical features of all *BGN* variant-harboring individuals (*n* = 54) from extended Meester-Loeys syndrome cohort

Organ system	Clinical feature	Frequency total	% total	Frequency males	% males	Frequency females	% females
Cardiovascular	Aortic root aneurysm	19/54	35%	14/25	56%	5/29	17%
	Dilated ascending aorta	6/42	14%	5/19	26%	1/23	4%
	Aortic dissection	4/49	8%	3/22	14%	1/27	4%
	Arterial aneurysm	9/36	25%	7/16	44%	2/20	10%
	Arterial dissection	4/32	13%	4/13	31%	0/19	0%
	Mitral valve prolapse	3/30	10%	2/11	18%	1/19	5%
	Hypertension	9/33	27%	1/11	9%	8/22	36%
Skeletal	Pectus deformity	7/45	16%	5/21	24%	2/24	8%
	Spine deformity	8/36	22%	5/14	36%	3/22	14%
	Dolichostenomelia	2/27	7%	1/10	10%	1/17	6%
	Arachnodactyly	8/40	20%	6/20	30%	2/20	10%
	Brachydactyly	6/37	16%	4/16	25%	2/21	10%
	Spatulous fingers	4/36	11%	3/16	19%	1/20	5%
	Flat feet	14/39	36%	9/19	47%	5/20	25%
	Club foot	1/30	3%	1/12	8%	0/18	0%
	Joint hypermobility	21/45	47%	12/21	57%	9/24	38%
	Joint dislocation	4/42	10%	3/18	17%	1/24	4%
	Joint contracture	8/43	19%	7/19	37%	1/24	4%
	Reduced bone density	6/13	46%	4/7	57%	2/6	33%
	Short stature	9/42	21%	7/21	33%	2/21	10%
	Tall stature	6/42	14%	5/21	24%	1/21	5%
Craniofacial	Dolichocephaly	5/37	14%	4/17	24%	1/20	5%
	Frontal bossing	5/37	14%	5/17	29%	0/20	0%
	Hypertelorism	9/44	20%	6/20	30%	3/24	13%
	Downslanting palpebral fissures	9/38	24%	6/17	35%	3/21	14%
	Proptosis	7/37	19%	4/17	24%	3/20	15%
	Malar hypoplasia	10/38	26%	7/18	39%	3/20	15%
	High-arched palate	5/34	15%	5/16	31%	0/18	0%
	Broad or bifid uvula	2/34	6%	2/17	12%	0/17	0%
	Gingival hypertrophy	2/24	8%	1/11	9%	1/13	8%
Ocular	Myopia	9/30	30%	4/12	33%	5/18	28%
Neuro-muscular	Mild learning problems	4/35	11%	4/16	25%	0/19	0%
	Dilated cerebral ventricles	4/15	27%	4/11	36%	0/4	0%
	Relative macrocephaly or large head circumference	7/20	35%	6/12	50%	1/8	13%
	Myopathy	1/29	3%	0/12	0%	1/17	6%
Cutaneous	Striae	7/38	18%	2/18	11%	5/20	25%
	Hypertrichosis	2/32	6%	2/14	14%	0/18	0%
	Delayed wound healing	3/33	9%	2/14	14%	1/19	5%
	Easy bruising	4/35	11%	1/15	7%	3/20	15%
	Umbilical hernia	2/36	6%	1/16	6%	1/20	5%

#### Cardiovascular features

Important cardiovascular features, all with a predominance in males, include aortic root aneurysm (35%; M/F: 56%/17%), ascending aortic aneurysm (14%; M/F: 26%/4%), and aortic dissection (8%; M/F: 14%/4%). Strikingly, 25% of the *BGN* variant-harboring individuals had arterial aneurysms (M/F: 44%/10%; Supplementary Fig. 2a, b) and 13% arterial dissections (M/F: 31%/0%). Both features occurred more frequently than originally described^1^, and showed a clear predominance in males.

#### Skeletal features

A wide range of skeletal features was observed in our MRLS cohort. Joint hypermobility (47%; M/F: 57%/38%), reduced bone density (46%; M/F: 57%/33%), and flat feet (36%; M/F: 47%/25%) were observed most often. Other recurrent skeletal features include pectus deformity, spine deformity, spatulous fingers, joint dislocation, and joint contractures. Both, short and tall statures were noted. Similarly, both, arachnodactyly and brachydactyly (Supplementary Fig. 2d) were observed.

#### Craniofacial features

The most common craniofacial features in our MRLS cohort include malar hypoplasia (26%; M/F: 39%/15%), downslanting palpebral fissures (24%; M/F: 35%/14%), and hypertelorism (20%; M/F: 30%/13%; Supplementary Fig. 2c). Other recurrent, but less frequent, craniofacial features were dolichocephaly, proptosis and gingival hypertrophy. Strikingly, broad or bifid uvula, highly arched palate, and frontal bossing were only noted in males.

#### Other features

Myopia was the only recurrent ocular feature, noted in 30% (M/F: 33%/28%). Mild learning problems (11%; M/F: 25%/0%) and ventriculomegaly (27%; M/F: 36%/0%) only occurred in males, while relative macrocephaly or large head circumference was noted for both sexes (35%; M/F: 50%/13%). Dermatomyositis was noted for one female proband. Regarding cutaneous manifestations, striae (18%; M/F: 11%/25%) and easy bruising (11%; M/F: 7%/15%) were observed more frequently in females than in males. Other cutaneous findings included hypertrichosis, delayed wound healing, and umbilical hernia.

## Discussion

In 2017, our research group first described loss-of-function variants in *BGN* as a genetic cause for an early-onset, syndromic form of TAAD^1^. The importance of biglycan in the pathogenesis of TAAD was substantiated by Heegaard et al.^13^ who described a male *Bgn*-knockout mouse model displaying spontaneous aortic dissections and ruptures before 12 weeks of age. Here, we characterize our extended MRLS cohort, consisting of 18 probands and 36 *BGN* variant-harboring family members, at a clinical and molecular level.

Regarding the clinical features, similarities between the initial publication^1^ and our extended cohort were observed, however, differences also became apparent. In the initial cohort, all five (male) probands, presented with either aortic root or ascending aortic aneurysms/dissections. The phenotype of female variant carriers, on the other hand, was considerably more variable, ranging from unaffected to death due to aortic dissection^1^. In our extended cohort, only 61% of the 18 probands presented with aortic root or ascending aortic involvement, with a higher incidence in males (63%) compared to females (50%). These data demonstrate a more variable expression of the aortic phenotype than initially described. Intriguingly, the opposite was true for arterial aneurysms and dissections, which were not yet recognized as key features of MRLS. While there were three individuals with an arterial aneurysm (pulmonary artery, patent ductus arteriosus, and brain) noted in the initial publication, 25% of the *BGN* variant carriers in this extended MRLS cohort presented with an arterial aneurysm, again with a predominance in males (44%) compared to females (10%). The two cohorts also differ in the prevalence of non-cardiovascular features. For instance, spatulous fingers, joint problems, and typical craniofacial features were more often described in the initial cohort^1^. Yet, pectus deformities, mild learning problems, delayed wound healing, easy bruising as well as umbilical hernia had similar frequencies as initially described^1^. The most recurrent clinical features in the current MRLS patient cohort, including both male and female *BGN* variant-harboring individuals, are aortic and arterial aneurysms, joint hypermobility, flat feet, malar hypoplasia, downslanting palpebral fissures, spine deformities, and short stature.

Due to the X-chromosomal localization of *BGN*, sexual dimorphism was anticipated. While 14 out of 16 probands were males (88%), 27 out of 36 variant-harboring family members were females (75%), confirming that males more often present with a clinical phenotype and females are predominantly identified through cascade screening. This is also supported by the lower overall incidence of clinical features in variant-harboring females compared to males.

Our in-depth molecular analysis demonstrates a loss-of-function mechanism as the underlying cause of MRLS. Identified *BGN* variants cause a stop codon insertion, frameshift, or splicing defect, which were either shown to lead to loss-of-function by cDNA and Western Blot analysis of patient skin fibroblasts or were strongly predicted to lead to loss-of-function based on the nature of the variant. Thus far, (likely) pathogenic missense variants were solely considered causal for MRLS if an additional (predicted) splice effect could be identified. Importantly, two missense variants in *BGN* that do not affect splicing (p.Lys147Gly and p.Gly259Val) have been reported to cause X-linked spondyloepimetaphyseal dysplasia (SEMDX, MIM #300106)^23^. It remains to be investigated what pathomechanisms are responsible for SEMDX development.

Remarkably, an identical frameshift variant was identified in families 7 and 8 (p.Asn148Lysfs*54). Although most *BGN* variants in our cohort are absent from GnomAD, this particular frameshift variant is seen three times in GnomAD (as two separate entries: rs782449715 and rs782199865)^22^. These three individuals are part of the European (Finnish) population. Since families 7 and 8 also originate from (Northern) Finland, we hypothesize that a founder mutation is underlying the higher prevalence of this variant in this region.

A distinct clinical feature, specifically mild skeletal dysplasia, was observed in males carrying a partial deletion of *BGN* and the 5’-UTR of the neighboring *ATP2B3*, compared to males with other loss-of-function *BGN* variants. Although loss-of-function of biglycan is determined to be the main pathomechanism underlying MRLS, additional mechanisms are hypothesized to explain the mild skeletal dysplasia on top of typical MRLS features in males carrying these specific deletions. RNA sequencing revealed that the remnant *BGN* 5’-UTR drives the expression of the neighboring *ATP2B3* because of this particular deletion. In healthy adults, *ATP2B3* is mainly expressed in brain tissues and the adrenal and pituitary glands (www.gtexportal.org). While considerable *ATP2B3* expression in skin fibroblasts was observed in the proband of family 17 carrying this particular deletion, no expression was seen in the skin fibroblasts of the control individuals. Although ectopic expression of *ATP2B3* was only investigated and observed in skin fibroblasts, we hypothesize that this phenomenon could occur in any tissue that typically expresses biglycan, including bone and cartilage. *ATP2B3* encodes plasma membrane Ca^2+^-transporting ATPase 3, which plays an important role in intracellular calcium homeostasis by exporting Ca^2+^ from the cytoplasm into the extracellular space to establish a Ca^2+^ gradient across the plasma membrane^24^. Since Ca^2+^ also plays an important role in bone homeostasis^25^, this ectopic *ATP2B3* expression could potentially explain the comorbid skeletal phenotype in these partial *BGN* and *ATP2B3* deletion carriers.

In conclusion, we describe an extended cohort of MRLS patients with hemizygous or heterozygous variants in *BGN*. In MRLS, aneurysms and dissections are not restricted to the level of the thoracic aorta but are also observed throughout the arterial tree, and cardiovascular features can be accompanied by non-specific connective tissue features. Furthermore, the clinical presentation is more severe and penetrant in males compared to females, although variable clinical expression has also been observed in males. Loss-of-function of biglycan is determined to be the underlying mutational mechanism in MRLS, which is corroborated by the complete loss of biglycan RNA and/or protein, or expression of an aberrant biglycan protein. Lastly, our molecular observations in partial *BGN* and *ATP2B3* deletion carriers represent a specific and distinct genotype-phenotype association for this disorder.

## Methods

### Human participants and DNA

This study is in accordance with the principles of the Declaration of Helsinki^26^, and was approved by the ethics committee of the Antwerp University Hospital (11/8/79). Written informed consent was provided by all study participants or their legally authorized representatives. Proband 3-I-2 also provided written informed consent for the publication of patient photographs. Patients with hemizygous or heterozygous variants in *BGN* were either identified in our diagnostic laboratory (Center of Medical Genetics, Antwerp University Hospital), or were referred to us by other laboratories, genetic centers, or GeneMatcher^27^. Clinical information was collected based on a standardized clinical checklist (Supplementary Methods). Z-scores for the aortic root and ascending aorta were calculated according to the formulas of Campens et al.^28^. DNA of affected and unaffected family members was requested whenever considered informative. Fibroblasts were cultured from skin biopsies of individuals from families 1, 6, 7, 9, 10, 11, 17, and 18, as well as male (1, 2, 3, and 4) and female (5 and 6) controls. *BGN* variants were classified based on the ACMG/AMP guidelines for sequence variant interpretation^29,30^. NM_001711.6 was used as a reference transcript for *BGN*, and GRCh38 was used as the genome reference build. Potential splice effects were investigated using the SpliceAI prediction score^31^ and with the Alamut™ Visual Plus software (version 1.4; SOPHiA GENETICS) which contains splicing prediction algorithms such as SpliceSiteFinder-like, MaxEntScan, NNSPLICE, and GeneSplicer.

### Cell culture

Skin fibroblasts were cultured in Roswell Park Memorial Institute (RPMI) medium (52400025, Gibco), supplemented with 15% fetal bovine serum (FBS; 10270106, Gibco), 1% sodium pyruvate (11360039, Thermo Scientific), 1% penicillin/streptomycin (15140122, Gibco) and 0.1% primocin (ant-pm-1, InvivoGen). Fibroblast cultures were incubated with and without puromycin (200 µg/mL, A1113803, Gibco) for 24 h to inhibit NMD.

### mRNA and cDNA sequencing

Skin fibroblasts were collected from the culture flask with TrypLE Express (12605010, Gibco), whereafter the cells were pelleted, and RNA was isolated using the Zymo Quick-RNA Miniprep kit (R1055, Zymo Research). RNA concentrations were determined with Nanodrop (Thermo Scientific), and RNA integrity numbers (RINs; ≥9.5) were defined using TapeStation 4150 (Agilent). Subsequent mRNA sequencing was outsourced to Novogene (Cambridge, United Kingdom). In short, the mRNA library was prepared using poly A enrichment, and the NovaSeq platform (Illumina) was used for 150 bp paired-end sequencing (9 Gb raw data/sample). Finally, the paired-end reads were aligned to GRCh38 with hisat2 version 2.0.5.

For cDNA sequencing, RNA extraction was followed by random hexamer cDNA conversion with the Superscript III First-Strand Synthesis kit for RT-PCR (18080051, Invitrogen). PCR was performed on the obtained cDNA. The PCR product was then purified using Alkaline Phosphatase (11097075001, Roche) and Exo I (M0293L, New England Biolabs). Purified PCR products were bidirectionally sequenced using the BigDye Terminator Cycle Sequencing kit (4462113, Applied Biosystems). The resulting sequencing product was purified using the CleanDTR paramagnetic bead-based system (CDTR-0005, CleanNA), and subsequently, separated on an ABI 3500xL Genetic Analyzer (Applied Biosystems). Sequences were analyzed with CLC DNA Workbench version 5 (CLC Bio). The PCR and sequencing primer sequences and reaction conditions are available upon request.

### Western blot

The medium was removed from the fibroblast cultures and the cells were washed with cold PBS. Then, proteins were isolated with 800 µL cold RIPA^+^ buffer (10 mL RIPA, 89900, Thermo Fisher Scientific; 1 tablet protease inhibitor cocktail, 11836170001, Roche; 1 tablet phosphatase inhibitors, 4906837001, Roche; 10 µL benzonase nuclease, 707464, EMD Millipore, Novagen). The cell lysate was gathered with a cell scraper and collected in an Eppendorf tube. The lysate was then incubated on a shaker on ice for 45 min and centrifuged at full speed for 5 min. The supernatant was transferred into a new tube.

Protein concentrations were determined using the Pierce BCA Protein Assay kit (23225, Thermo Fisher Scientific). Subsequently, the lysates were incubated with NuPage LDS sample buffer (NP0007, Invitrogen) and NuPage Reducing agent (NP0004, Invitrogen) at 70 °C for 10 min. Equal protein concentrations were subjected to gel electrophoresis using a Bis-Tris 4–12% mini gel (NP0322BOX or NW04125BOX; Invitrogen) and the PageRuler Plus Prestained Protein Ladder (26619, Thermo Fisher Scientific). Proteins were then transferred onto a 0.1 μm nitrocellulose membrane (10600000, Amersham Protran) and blocked in a 5% milk (5601001004797, Nestlé) in TBS-T solution for 2 h. Primary antibody incubation (BGN, AF2667, R&D systems, 1:500; Cyclophilin B, PA1-027A, Thermo Fisher Scientific, 1:1000) was performed at 4 °C overnight. Next, membranes were washed with TBS-T and incubated with the respective secondary antibody (rabbit anti-goat IgG, 1:10,000, 31402, Invitrogen; goat anti-rabbit IgG, 1:10,000, 1706515, Bio-Rad) for 2 h at RT. Pierce^TM^ ECL or SuperSignal™ West Femto Western Blotting reagents (32106 or 34095, Thermo Fisher Scientific) were used as detection substrates. Images were acquired with an ImageQuant LAS 4000 Mini (Cytiva), and protein signals were analyzed using ImageJ (National Institutes of Health). Cyclophilin B was used as a loading control. Uncropped images are supplied in Supplementary Figs. 3 and 4.

### Reporting summary

Further information on research design is available in the Nature Research Reporting Summary linked to this article.

### Supplementary information

Reporting Summary

Supplementary Information

## Data Availability

Due to restrictions in the informed consent sharing of data in secure access-controlled repositories is not allowed but for qualified researchers data of this study are available from the corresponding author upon reasonable request. The ClinVar accession numbers for the *BGN* variants reported in this study are SCV004170959 to SCV004170969, VCV000265797.1, and VCV000265798.2.

## Code availability

The code that was used to analyze mRNA sequencing data is available upon reasonable request.
