# Supplementary material for: Expanding the clinical spectrum of biglycan-related Meester-Loeys syndrome
Source: NPJ Genom Med. 2024 Mar 26;9:22. doi: 10.1038/s41525-024-00413-z (PMC10966070; doi:10.1038/s41525-024-00413-z)
Supplement: Supplementary file 1 — Reporting Summary [file 41525_2024_413_MOESM1_ESM.pdf]

# nature portfolio

Corresponding author(s): B. L. Loeys

Last updated by author(s): Mar 7, 2024



# Reporting Summary

Nature Portfolio wishes to improve the reproducibility of the work that we publish. This form provides structure for consistency and transparency in reporting. For further information on Nature Portfolio policies, see our Editorial Policies and the Editorial Policy Checklist.

## Statistics

For all statistical analyses, confirm that the following items are present in the figure legend, table legend, main text, or Methods section.

| n/a | Confirmed | |
|---|---|---|
| ☒ | ☐ | The exact sample size (*n*) for each experimental group/condition, given as a discrete number and unit of measurement |
| ☒ | ☐ | A statement on whether measurements were taken from distinct samples or whether the same sample was measured repeatedly |
| ☒ | ☐ | The statistical test(s) used AND whether they are one- or two-sided<br>*Only common tests should be described solely by name; describe more complex techniques in the Methods section.* |
| ☒ | ☐ | A description of all covariates tested |
| ☒ | ☐ | A description of any assumptions or corrections, such as tests of normality and adjustment for multiple comparisons |
| ☒ | ☐ | A full description of the statistical parameters including central tendency (e.g. means) or other basic estimates (e.g. regression coefficient) AND variation (e.g. standard deviation) or associated estimates of uncertainty (e.g. confidence intervals) |
| ☒ | ☐ | For null hypothesis testing, the test statistic (e.g. *F*, *t*, *r*) with confidence intervals, effect sizes, degrees of freedom and *P* value noted<br>*Give P values as exact values whenever suitable.* |
| ☒ | ☐ | For Bayesian analysis, information on the choice of priors and Markov chain Monte Carlo settings |
| ☒ | ☐ | For hierarchical and complex designs, identification of the appropriate level for tests and full reporting of outcomes |
| ☒ | ☐ | Estimates of effect sizes (e.g. Cohen's *d*, Pearson's *r*), indicating how they were calculated |

*Our web collection on statistics for biologists contains articles on many of the points above.*

## Software and code

Policy information about availability of computer code

| | |
|---|---|
| Data collection | No software was used to collect data. |
| Data analysis | The code that was used to analyze mRNA sequencing data is available upon reasonable request. |

For manuscripts utilizing custom algorithms or software that are central to the research but not yet described in published literature, software must be made available to editors and reviewers. We strongly encourage code deposition in a community repository (e.g. GitHub). See the Nature Portfolio guidelines for submitting code & software for further information.

## Data

Policy information about availability of data

All manuscripts must include a data availability statement. This statement should provide the following information, where applicable:

- Accession codes, unique identifiers, or web links for publicly available datasets
- A description of any restrictions on data availability
- For clinical datasets or third party data, please ensure that the statement adheres to our policy

Data that support the findings of this study are available from the corresponding author upon reasonable request. The ClinVar accession numbers for the BGN variants reported in this study are SCV004170959 to SCV004170969, VCV000265797.1 and VCV000265798.2.

## Research involving human participants, their data, or biological material

Policy information about studies with human participants or human data. See also policy information about sex, gender (identity/presentation), and sexual orientation and race, ethnicity and racism.

| | |
|---|---|
| Reporting on sex and gender | Findings apply to sex.<br>Individuals of both sexes were included in the study.<br>Sex was determined by the referring clinician and confirmed by DNA sequencing data.<br>Our MRLS cohort consists of 18 probands (16 males and 2 females) and 36 BGN variant-harboring family members (9 males and 27 females).<br>Differences in clinical features were compared between sexes. |
| Reporting on race, ethnicity, or other socially relevant groupings | There were no socially constructed or socially relevant categorization variables used. |
| Population characteristics | Pedigree, genotypic information, sex, age at onset, age of death, clinical data |
| Recruitment | Patients with hemizygous or heterozygous variants in BGN were either identified in our diagnostic laboratory (Center of Medical Genetics, Antwerp University Hospital), or were referred to us by other laboratories, genetic centers or GeneMatcher.<br>Since Meester-Loeys syndrome is an ultra-rare disease, an inherent selection bias due to, for instance, missed genetic or phenotypic diagnoses, variable clinical expression, lack of knowledge is likely to exist. This inherent bias to rare disease research does not impact the validity of our study, it will rather decrease selection bias in the future due to raised awareness. |
| Ethics oversight | Ethics committee of the Antwerp University Hospital (11/8/79). |

Note that full information on the approval of the study protocol must also be provided in the manuscript.

# Field-specific reporting

Please select the one below that is the best fit for your research. If you are not sure, read the appropriate sections before making your selection.

☒ Life sciences ☐ Behavioural & social sciences ☐ Ecological, evolutionary & environmental sciences

For a reference copy of the document with all sections, see nature.com/documents/nr-reporting-summary-flat.pdf

# Life sciences study design

All studies must disclose on these points even when the disclosure is negative.

| | |
|---|---|
| Sample size | No sample size calculation was performed. |
| Data exclusions | No data was excluded. |
| Replication | Attempts at replication were successful. |
| Randomization | This is not relevant to our study since there are no experimental groups. |
| Blinding | Blinding was not possible since the investigators were also designing and performing the experiments. |

# Reporting for specific materials, systems and methods

We require information from authors about some types of materials, experimental systems and methods used in many studies. Here, indicate whether each material, system or method listed is relevant to your study. If you are not sure if a list item applies to your research, read the appropriate section before selecting a response.

## Materials & experimental systems

| n/a | Involved in the study |
| --- | --- |
| ☐ | ☒ Antibodies |
| ☐ | ☒ Eukaryotic cell lines |
| ☒ | ☐ Palaeontology and archaeology |
| ☒ | ☐ Animals and other organisms |
| ☒ | ☐ Clinical data |
| ☒ | ☐ Dual use research of concern |
| ☒ | ☐ Plants |

## Methods

| n/a | Involved in the study |
| --- | --- |
| ☒ | ☐ ChIP-seq |
| ☒ | ☐ Flow cytometry |
| ☒ | ☐ MRI-based neuroimaging |

# Antibodies

| | |
| --- | --- |
| Antibodies used | BGN (R&D systems, AF2667, polyclonal goat IgG, lot VGM0420011).<br>CypB (Thermo Fisher Scientific, PA1-027A, polyclonal rabbit IgG, lot XG354148).<br>Rabbit anti-goat IgG (Invitrogen, 31402).<br>Goat anti-rabbit IgG (Bio-Rad, 1706515). |
| Validation | The BGN antibody detects biglycan in human tissues and was validated by the manufacturer with ELISAs and Western Blots. We confirmed the validity by including a negative control (MRLS patient with a biglycan deletion).<br>The CypB antibody detects cyclophilin B in mouse, rat, canine, and human tissues. It was validated by the manufacturer with Western blot procedures, IF/ICC and IHC. Additionally, this antibody was verified by Knockdown to ensure that the antibody binds to the antigen stated. |

# Eukaryotic cell lines

Policy information about cell lines and Sex and Gender in Research

| | |
| --- | --- |
| Cell line source(s) | Fibroblasts were cultured from skin biopsies of individuals from families 1 (one male), 6 (one male), 7 (one male, one female), 9 (one male), 10 (one male), 11 (one male), 17 (one male) and 18 (one male), as well as controls (four males, two females). |
| Authentication | None of the cell lines were authenticated |
| Mycoplasma contamination | Cell lines were not tested for mycoplasma contamination |
| Commonly misidentified lines<br>(See ICLAC register) | No commonly misidentified cell lines were used in the study. |

# Plants

| | |
| --- | --- |
| Seed stocks | *Report on the source of all seed stocks or other plant material used. If applicable, state the seed stock centre and catalogue number. If plant specimens were collected from the field, describe the collection location, date and sampling procedures.* |
| Novel plant genotypes | *Describe the methods by which all novel plant genotypes were produced. This includes those generated by transgenic approaches, gene editing, chemical/radiation-based mutagenesis and hybridization. For transgenic lines, describe the transformation method, the number of independent lines analyzed and the generation upon which experiments were performed. For gene-edited lines, describe the editor used, the endogenous sequence targeted for editing, the targeting guide RNA sequence (if applicable) and how the editor was applied.* |
| Authentication | *Describe any authentication procedures for each seed stock used or novel genotype generated. Describe any experiments used to assess the effect of a mutation and, where applicable, how potential secondary effects (e.g. second site T-DNA insertions, mosiacism, off-target gene editing) were examined.* |
