# Supplementary material for: Expanding the clinical spectrum of biglycan-related Meester-Loeys syndrome
Source: NPJ Genom Med. 2024 Mar 26;9:22. doi: 10.1038/s41525-024-00413-z (PMC10966070; doi:10.1038/s41525-024-00413-z)
Supplement: Supplementary file 2 — Supplementary Information [file 41525_2024_413_MOESM2_ESM.pdf]

SUPPLEMENTARY INFORMATION

Supplementary Figure 1 2
Supplementary Table 1 3
Supplementary Notes 5
Supplementary Figure 2 9
Supplementary Methods 10
Supplementary Figure 3 13
Supplementary Figure 4 14

Supplementary Figure 1

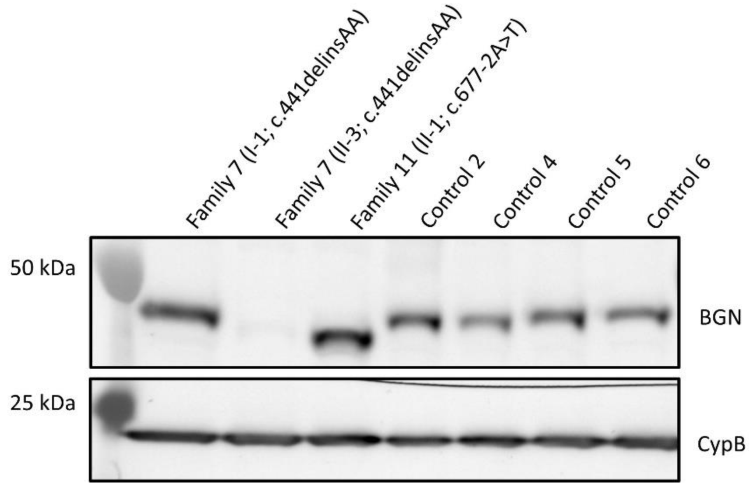

Supplementary Figure 1. Western Blot of BGN protein expression in skin fibroblasts of *BGN* variant carriers of families 7 and 11 as well as controls. Intracellular proteins were isolated from available skin fibroblast samples and the biglycan (BGN) protein content was visualized. Cyclophilin B (CypB) was used as a loading control. Control 2 and 4 were samples of age-matched males, and Control 5 and 6 were samples of age-matched females. The Western Blot was derived from one experiment and all lanes were processed in parallel.

Supplementary Table 1

Family ID	c. notation	p. notation	ID	Proband	Mutation status	Sex	Age at onset	Age of death	cDNA analysis	Protein analysis	Other variant information	Cardiovascular								Skeletal										
												Aortic root aneurysm	Aorta ascendens aneurysm	Aortic dissection	Arterial aneurysm	Arterial dissection	Mitral valve prolapse	Hypertension	Other	Pectus deformity	Spine deformity	Dolicho-stenomelia	Arachno dactyly	Brachy-dactyly	Spatulous fingers	Flat feet	Club foot	Joint hypermobility	Joint dislocation	Joint contracture
1	c.46delG	p.Ala16Profs*20	1-III-3	Yes	Hemizygous	M	37	-	No NMD.	Complete loss	-	+ (43 mm)	+ (38 mm)	-	common, external, and internal iliac arteries	common, external, and internal iliac arteries, truncus coeliacus, arteria hepatica communis, arteria lienalis and renal artery	-	-	arteria lusoria, intramural hematoma in common iliac artery, retroperitoneal hematoma, hematoma in paracolic gutter and hematoma in pouch of Douglas	-	-	-	+/-	-	-	-	-	+	-	-
			1-II-4	No	Heterozygous	F	-	-	-	-	-	-						+	-	-	-							-	-	-
			1-II-2	No	Heterozygous	F	-	-	-	-	-	-	-	-	-	-		+	elongated, narrow aorta	-	-							-	-	-
			1-II-3	No	Heterozygous	F	-	-	-	-	-	-	-	-	-	-		+	narrow aorta and stenosis of subclavian artery	-	-							-	-	-
			1-III-2	No	Hemizygous	M	-	-	-	-	-	-	-	-	-	-		-	narrow aorta and arteries	-	-							-	-	-
			1-IV-1	No	Heterozygous	F	-	-	-	-	-	-	-	-			-	-	-	-	-	-	-	-	-	-	-	+	-	-
			1-IV-2	No	Heterozygous	F	-	-	-	-	-	-	-	-			-	-	-											
2	c.59_60insAA	p.Gln21Serfs*16	2-II-1	Yes	Hemizygous	M	66	-	-	-	-	+ (60 mm)						-	myxoma near foramen ovale		kyphosis									Dupuytren's contracture in both hands
3	c.75G>A	p.Trp25*	3-I-2	Yes	Hemizygous	M	61	-	-	-	-	-	-	-	bilateral iliac arteries, coeliac trunk, superior mesenteric artery, renal arteries	superior mesenteric artery			venous insufficiency	-	-		-	+	-	+	-	+	-	-
			3-II-2	No	Obligate carrier	F	30	-	-	-	-	-	-	-	-	-	-	-	lymphoedema of the lower limbs	-	-	-	-	-	-	-	-	Fingers	-	-
4	c.75G>A	p.Trp25*	4-III-3	Yes	Hemizygous	M	0	-	-	-	Maternally inherited VUS in OPHN1 (NM_002547.3) for hydrocephalus: c.1025+13G>A	-	-	-	-	-	-	-	small patent foramen ovale with predominantly left to right shunt, redundant mitral valve, and mild dilatation of right ventricle	excavatum	-	-	-	-	-	-	-	-	-	-
			4-II-2	No	Heterozygous	F	-	-	-	-	VUS in OPHN1 (NM_002547.3): c.1025+13G>A	-	-	-	-	-	-/+	-	-	-	scoliosis	-	-	-	-	-	-	-	-	-
5	c.223C>T	p.Gln75*	5-II-2	Yes	Heterozygous	F	-	-	-	-	-	-	-	-	-	-	-	-	-	-	-		-	-	-	-	-	Elbows	-	-
			5-III-1	No	Heterozygous	F	-	-	-	-	-	-	-	-					-									+	-	-
			5-I-1	No	Heterozygous - reported by proband	F	-	-	-	-	-								-											
6	c.351+1G>A	p.Tyr117_Ala118insIleArg-SerTrpGluGluProAlaGlyLeu-GlnGlnArgAlaGlyValArg	6-III-1	Yes	Hemizygous	M	0	-	Incorporation of 51 nucleotides of intron 3 (in-frame, 17 amino acids, no stop codon). No NMD.	No loss, longer protein	-	-	-	-	-	-	-	-	-	-	+	-	-	-	-	+	+	-	-	+
			6-II-2	No	Heterozygous	F	0	-	-	-	-	-	-	-	-	-	-	+	-	-	+	-	-	-	-	+	-	-	-	+
			6-II-3	No	Heterozygous	F	71	-	-	-	-	-	-	-	-	-	-	+	tricuspid aortic valve	-	-	-	-	-	-	+	-	Fingers	-	-
			6-III-4	No	Heterozygous	F	30	-	-	-	-	-	-	-	-	-	-	+	-	-	-	long legs	-	-	-	-	-	Knees and hips	-	-
7	c.441delinsAA	p.Asn148Lysfs*54	7-II-3	Yes	Hemizygous	M	11	-	For the vast majority of transcripts, NMD is seen.	Complete loss	Heterozygous *de novo* VUS in *RORA* (NM_134261.2): c.1283T>C, p.Leu428Pro	-	-	-	-	-	-	-	-	-	-	-	-	-	-	+	-	Bilateral thumb, left elbow, right knee.	-	-
			7-I-1	No	Heterozygous	F	-	-	For a fraction of the transcripts, the frameshift is detected. No NMD. The wildtype allele dominates.	Wildtype	-	-	-	-	-	-	-	+	migraine	-	-	-	-	-	-	-	-	-	-	-
8	c.441delinsAA	p.Asn148Lysfs*54	8-II-2	Yes	Hemizygous	M	70	-	-	-	-	-	-	-	left circumflex femoral artery aneurysm (36 mm), right circumflex femoral artery aneurysm (27 mm), right popliteal artery aneurysm (16 mm), left common iliac artery aneurysm (32 mm)	-	+	+	peripheral arterial disease, slight dilation of upper descending thoracic aorta (37 mm) and coronary artery disease	-	scoliosis	-	-	-	-	+	-	-	-	-
			8-II-1	No	Obligate carrier	M	-	30	-	-	-															+				
			8-III-2	No	Heterozygous	F	34	-	-	-	-	-	-	-	-	-	-	-	-	excavatum	-	-	+/-	-	-	+	-	Bilateral thumb apposition to forearm and palms to floor.	-	-
			8-III-1	No	Heterozygous	F	37	-	-	-	-	-	-	-	-	-	-	-	-	-	-	-	+/-	-	-	+	-	Left thumb apposition to forearm and palms to floor.	-	-
9	c.565G>A	p.Val174Argfs*20 (or p.Glu189Lys)	9-II-3	Yes	Hemizygous	M	54	-	Alternative splicing in approximately 50% of the transcripts. Alternatively spliced transcript has 46 nucleotides deleted (out-of-frame) and partly undergoes NMD. The wildtype spliced transcript contains the missense variant (Gly189Lys).	Complete loss	Relatively common VUS in *LRP5* : c.3107G>A, p.Arg1036Gln	-	-		right vertebral artery, renal arteries, basilar trunk	left vertebral artery			-											
			9-II-4	No	Hemizygous	M	-	-	-	-	-	-	-						-											
			9-I-5	No	Obligate carrier	F	-	76	-	-	-								-											
10	c.677-2A>G	p.Asp225_Leu236del	10-II-2	Yes	Hemizygous	M	51	-	Shift from canonical to cryptic splice site, leading to a deletion of 33 nucleotides (in-frame, 11 amino acids). No NMD.	No loss, shorter protein	*SLC2A10* variant carriership	+ (80 mm)	+ (40 mm)	-	-	renal artery	-	-	bicuspid aortic valve with limited insufficiency, calcifications and stenosis, aneurysm of left coronary sinus and cardiomyopathy (left ventricular ejection fraction of 40%)	excavatum	-	-	-	-	-	-	-	-	-	-
			10-II-1	No	Hemizygous	M	56	-	-	-	-	-	-	-	-	-	-	-	-	-	-	-	-	-	-	-	-	-	-	-
			10-II-3	No	Heterozygous	F	51	-	-	-	-	+ (36 mm)	-	-	millimetric infundibular widening of a branch of anterior ACM sinistra	-	-	-	-	-	-	-	-	-	-	-	-	-	-	-
			10-I-1	No	Obligate carrier	F	-	58	-	-	-	-	-	-	-	-	-	+	-	-	-	-	-	-	-	-	-	-	-	-
11	c.677-2A>T	p.Asp225_Leu236del	11-II-1	Yes	Hemizygous	M	43	-	Shift from canonical to cryptic splice site, leading to a deletion of 33 nucleotides (in-frame, 11 amino acids. No NMD.	No loss, shorter protein	Likely pathogenic truncating variant in *TRPS1* (NM_014112.4):c.3252C>A, p.Tyr1084*	+ (45 mm)	-	-	celiac, mesenteric, hepatic, femoral, popliteal and tibioperoneal arteries				chronic ischemia but normal vessels	-			-	+	-	-	-	+	-	-
12	c.770+1G>A	p.?	12-II-1	Yes	Hemizygous	M	25	29	-	-	-	+	+	+	-	-	-		ventricular septal defect	+	scoliosis	+	+	-	-	+	-	+	+	+
			12-I-2	No	Heterozygous	F	-	-	-	-	-	-	-	-	-	-	-	-	-	-	-	-	-	+	+	-	-	+	-	-
			12-II-3	No	Not tested	F	13	-	-	-	-	-	-	-	-	-	-	-	postural orthostatic tachycardia syndrome	-	-	+	-	-	-	-	-	+	-	-
13	c.910-1G>A	p.?	13-III-2	Yes	Heterozygous	F	59	-	-	-	-	-	+ (41 mm)	-	-	-	-	+	common origin of brachiocephalic artery and left common carotid artery	+	-	-	-	-	-	-	-	-	-	-
			13-II-2	No	Obligate carrier	F	68	73	-	-	-	+	+	-	-	-			-		kyphosis									
			13-IV-2	No	Heterozygous	F	-	-	-	-	-	-	-	-	-	-	-	-	-	-	-	-	-	-	-	-	-	-	-	-
			13-IV-4	No	Hemizygous	M	35	-	-	-	-	-	-	-	-	-	-	-	-	-	-	-	-	-	-	-	-	-	-	-
			13-IV-7	No	Heterozygous	F	-	-	-	-	-	-	-	-	-	-	-	-	-	-	-	-	-	-	-	-	-	-	-	-
			13-V-2	No	Heterozygous	F	-	-	-	-	-	-	-	-	-	-	-	-	-	-	-	-	-	-	-	-	-	-	-	-
			13-V-6	No	Heterozygous	F	-	-	-	-	-	-	-	-	-	-	-	-	-	-	-	-	-	-	-	-	-	-	-	-
			13-V-7	No	Heterozygous	F	-	-	-	-	-	-	-	-	-	-	-	-	-	-	-	-	-	-	-	-	-	-	-	-
			13-V-8	No	Hemizygous	M	-	-	-	-	-	-	-	-	-	-	-	-	-	-	-	-	-	-	-	-	-	-	-	-
			13-V-13	No	Heterozygous	F	-	-	-	-	-	-	-	-	-	-	-	-	-	-	-	-	-	-	-	-	-	-	-	-

				Skeletal			Craniofacial										Ocular		Neuromuscular					Cutaneous						
Family ID	c. notation	p. notation	ID	Reduced bone density	Stature	Other	Dolichocephaly	Hypertelorism	Down-slanting eyes	Broad or bifid uvula	Highly arched palate	Proptosis	Malar hypoplasia	Frontal bossing	Gingival hypertrophy	Other	Myopia	Other	Mild learning problems	Dilated cerebral ventricles	Relative macrocephaly	Myopathy	Other	Striae	Hypertrichosis	Delayed wound healing	Easy bruising	Umbilical hernia	Other	Other
1	c.46delG	p.Ala16Profs*20	1-III-3	osteopenia	tall (193 cm)	-	-	-	-	-	-	-	-	-	-	-	-	-	-			-	-	-	-	-	-	-	inguinal hernia in childhood	mesenteric lymph nodes of slightly progressive nature
			1-II-4		normal (163 cm)	-										-		-					-						-	-
			1-II-2		tall (177 cm)	-		-								-		-					-						-	-
			1-II-3		normal (170 cm)	-		-								-		-					-					+	-	gallstones and anterior cutaneous nerve entrapment syndrome
			1-III-2		tall (193 cm)	-										-		-					-						-	-
			1-IV-1		normal (94 cm)	endorotation of both feet	-	-	-							-	-	-					-	-					-	-
			1-IV-2		normal (110 cm)	-										-		-					-						-	-
2	c.59_60insAA	p.Gln21Serfs*16	2-II-1		normal (186 cm)	syndactyly between 2nd and 3rd toe										-	+	-			large head circumference (60cm)		-						-	hearing loss
3	c.75G>A	p.Trp25*	3-I-2	-	normal (176 cm)	-	-	-	-	-	+	-	-	-	-	-	-	-	-	-	-	-	-	-	-	+	-	-	soft skin lacking elasticity (cutis laxa-like)	-
			3-II-2	-	normal (164 cm)	-	-	-	-	-	-	-	-	-	-	-	-	-	-	-	-	-	-	-	-	-	-	-	-	-
4	c.75G>A	p.Trp25*	4-III-3		normal (55 cm)	mild 2-4 toe syndactyly	-	+	+	-	-	-	-	-	-	-		-	-	+	+	-	hydrocephaly	-	-		-	-	-	-
			4-II-2	-	normal (160 cm)	-	-	-	-	-	-	-	-	-	-	-	-	-	-	-	-	-	-	+	-	-	-	-	-	irritable bowel syndrome
5	c.223C>T	p.Gln75*	5-II-2		normal (169 cm)	-	-	-	-	-	-	-	-	-	-	-	-	-	-		large head circumference (55cm)	-	-	pregnancy-related	-	-	-	-	-	-
			5-III-1			dyspraxia		-	-		-		-	-	-	-	-	-	-				-	-			+	-	-	-
			5-I-1			-										-		-					-						-	-
6	c.351+1G>A	p.Tyr117_Ala118insIleArg-SerTrpGluGluProAlaGlyLeu-GlnGlnArgAlaGlyValArg	6-III-1		tall (192 cm)	Beals Syndrome, camptodactyly and joint problems	-	-	-	-	-	-	-	-	-	unilaterally abnormal earlobe of crumpled appearance as baby	-	-	-	-	-	-	-	-	-	-	-	-	-	-
			6-II-2	osteopenia	normal (170 cm)	congenital hammer toes	-	-	-	-	-	-	-	-	-	-	-	-	-	-	-	-	-	-	-	-	-	-	-	hypercholesterolemia
			6-II-3		normal (170 cm)	moderate degenerative changes in C4-7 spine	-	-	-	-	-	-	+	-	-	-	+	-	-			-	-	-	-	-	-	-	-	-
			6-III-4	-	normal (167 cm)	self-reported back pain	+	-	-			-	+	-	-	-	+	-	-	-	-	-	-	-	-	-	-	-	-	right lobe or thyroid lesion
7	c.441delinsAA	p.Asn148Lysfs*54	7-II-3		short (131 cm)	joint pain	-	-	-	-	+	-	-	-	-	-	-	-	+	-	-	hypotonia until 1.5y	sensory defensiveness, and dyslexia and methylphenidate medication to improve concentration and alertness	-	-	+	-	-	-	Removal of congenital nevus from the right shoulder, asthma, atopic rash, and allergic rhinitis
			7-I-1		normal (164 cm)	-	-	-	-	-	-	-	-	-	-	-		-	-		-	-	-	-	-	-	-	-	-	-
8	c.441delinsAA	p.Asn148Lysfs*54	8-II-2		normal (180 cm)	multiple fractures and mild skin hyperextensibility	-	-	+	-	-	-	+	-	-	-	+	astigmatism	-	-	-	-	-	-	-	-	-	-	-	-
			8-II-1		normal (182 cm)											-		-					-						-	-
			8-III-2		normal (171 cm)	mild skin hyperextensibility	-	-	-	-	-	-	-	-	-	low-set posteriorly rotated ears	+	astigmatism	-		-	-	-	-	-	-	-	-	-	-
			8-III-1		normal (166 cm)	mild skin hyperextensibility	-	-	-	-	-	-	-	-	-	-	-	-	-		-	-	-	+	-	-	+	-	-	-
9	c.565G>A	p.Val174Argfs*20 (or p.Glu189Lys)	9-II-3	osteoporosis		multiple fractures										-		-					-						-	-
			9-II-4	osteoporosis		-										-		-					-						-	bleeding disorder history
			9-I-5			-										-		-					-						-	died of hepatic cancer and had a bleeding disorder history
10	c.677-2A>G	p.Asp225_Leu236del	10-II-2	osteoporosis	normal (184 cm)	-	-	-	-	-	-	-	-	-	-	-	+	-	-	-	-	-	-	-	-	-	+	-	-	hypercholesterolemia and recurrent pneumothorax
			10-II-1	-	short (168 cm)	-	-	-	-	-	-	-	-	-	-	-	-	-	-	-	-	-	-	-	-	-	-	-	-	-
			10-II-3	osteopenia	short (163 cm)	-	-	-	-	-	-	-	-	-	-	-	+	retinitis pigmentosa with complete blindness	-	-	-	-	-	-	-	-	-	-	-	-
			10-I-1	-	tall (180 cm)	-	-	-	-	-	-	-	-	-	-	-	+	-	-	-	-	-	-	-	-	-	-	-	-	diabetes/obesity/hypercholesterolemia/ASHD (PCI)
11	c.677-2A>T	p.Asp225_Leu236del	11-II-1	-	short (160 cm)	bilateral avascular necrosis of the hip	-	-	-	-	-	+	-	-	-	mild ptosis, round face and pointed nasal tip	-	keratoconus	-	-	+	-	-	-	-	-	-	-	-	-
12	c.770+1G>A	p.?	12-II-1		tall (196 cm)	-	-	-	-		+	-	-	-	-	-	+	-	+			-	-	-	-	-	-	-	-	-
			12-I-2	-	normal (170 cm)	-	-	-	+	-	-	-	-	-	-	-	+	-	-			-	-	-	-	-	-	-	-	type 2 diabetes mellitus
			12-II-3	-	tall (177 cm)	-	-	-	-	-	+	-	-	-	-	-	+	-	-			-	-	+	-	-	+	-	-	polycystic ovary syndrome
13	c.910-1G>A	p.?	13-III-2	-	normal (158 cm)		-	-	-	-	-	+	-	-			-		-			dermatomyositis			-	-	-			
			13-II-2	osteoporosis																										
			13-IV-2		normal (163 cm)		-	-	-	-	-	-	-	-			-		-			-			-	-	-	-		
			13-IV-4		normal (180 cm)		-	-	-	-	-	-	-	-			-		+			-		-	-	-	-	-	Inguinal hernia	
			13-IV-7		normal (160 cm)		-	-	-	-	-	-	-	-			-		-			-		+	-	-	-	-		
			13-V-2				-	-	-	-	-	-	-	-			-		-			-		-	-	-	-	-		
			13-V-6				-	-	-	-	-	-	-	-			-		-			-		-	-	-	-	-		
			13-V-7				-	-	-	-	-	-	-	-			-		-			-		-	-	-	-	-		
			13-V-8				-	-	-	-	-	-	-	-			-		-			-		-	-	-	-	-		
			13-V-13				-	-	-	-	-	-	-	-			-		-			-		-	-	-	-	-		

Supplementary Notes

Family 1 – The male proband of family 1 (1-III-3, 40 y) had multiple dissections of smaller intraabdominal arteries (truncus coeliacus extending in hepatic and spleen artery; left renal artery; left iliac artery extending into external and internal iliac artery). The maximum aortic root diameter was 43 mm at the age of 38, corresponding with a z-score of 2.92. The proximal ascending aorta had a diameter of 38 mm, which corresponds with a z-score of 2.54. No thickening of the aortic wall and no aortic dissections were observed. The proband had joint hypermobility and generalized osteopenia. He had a tall stature (193 cm). The brother (1-III-2, 36 y) of the proband had a narrow aorta and arteries, but no aneurysms or signs of dissections on magnetic resonance angiography (MRA). He also had a tall stature (193 cm). No cardiovascular abnormalities on echocardiography were observed in the daughters of the proband (1-IV-1, 5 y and 1-IV-2, 7 y). Daughter 1-IV-5 presented with joint hypermobility and endorotation of both feet. She had a normal stature (94 cm). The MRA of an aunt (1-II-3, 63 y) showed a stenosis of the subclavian artery. No aneurysms or signs of dissections were detected. She had hypertension, gallstones, anterior cutaneous nerve entrapment syndrome and a normal stature (170 cm). The MRA of another aunt (1-II-2, 64 y) showed an elongated, narrow aorta without aneurysms or signs of dissections. She had hypertension and a tall stature (177 cm). Segregation analysis confirmed the presence of the *BGN* variant in these six family members.

Family 2 – The male proband (2-II-1) was diagnosed with aortic root dilatation at age 66 (60 mm, z-score of 5.64), upon which he underwent surgery. He had a myxoma in the heart in the region of the foramen ovale, which was removed during the same procedure. The proband had myopia (approximately -1.5 dioptres) and hearing loss. He had Dupuytren's contracture in both hands since childhood, which was operated but was recurring. The patient's height was 185.8 cm with a head circumference of 60 cm. He presented with mild kyphosis, brachydactyly and syndactyly between the second and third toes of both feet. The patient's brother (2-II-2) died due to a rupture of a brain aneurysm at age 36.

Family 3 – The male proband (3-I-2) was 176 cm and weighed 83 kg at age 61. He had multiple arterial aneurysms at the following locations: bilateral iliac arteries, coeliac trunk, superior mesenteric artery, and renal arteries. The aneurysm of the superior mesenteric artery dissected. The aorta measured 36 mm at sinus of Valsalva (z-score of 0.16). No craniofacial features were observed, except for a highly arched palate. He had joint hypermobility, flat feet, brachydactyly, camptodactyly secondary to a Dupuytren's disease, delayed wound healing and venous insufficiency. He had soft skin with a cutis laxa-like appearance. His daughter (3-II-2) is an obligate carrier of the *BGN* variant. Her aortic root measured 30 mm (z-score of 0.83). She presented with lymphoedema of the lower limbs, joint hypermobility of the fingers, and normal stature.

Family 4 – The male proband (4-III-3, 0.5 y) was born at 38 weeks with a length of 54 cm (98th percentile, z-score of 2.1), weight of 4.59 kg (99th percentile, z-score of 2.3) and head circumference of 44.5 cm (>99th percentile, z-score of 7.9). He presented with marked macrocephaly, hypertelorism, downslanting palpebral fissures, pectus excavatum and 2-4 toe syndactyly. Brain imaging showed marked ventriculomegaly. Echocardiography demonstrated small patent foramen ovale with predominantly left to right shunt, normal aortic diameters, slight dilation of the right ventricle but preserved left and right ventricular function. At four months, he was status post ventriculoperitoneal shunt and was doing well, meeting developmental milestones. Macrocephaly had improved to 45 cm (99th percentile, z-score of 2.5). This proband inherited the *BGN* variant as well as a VUS in *OPHN1* for hydrocephalus[32-34] (NM_002547.3: c.1025+13G>A) from his clinically healthy mother (4-II-2, 39 y). She was 160 cm tall and weighed 68 kg. No craniofacial features were observed. Notably, she presented with scoliosis, striae, borderline mitral valve prolapse and irritable bowel syndrome. Echocardiography performed shortly after diagnosis revealed borderline prolapse of the anterior mitral valve leaflet and mild mitral regurgitation. Complete angiography revealed no aneurysmal dilatation of the thoracic and abdominal aorta or iliac vessels.

Family 5 – In the female proband (5-II-2, 44 y), the *BGN* variant was detected as part of a comprehensive prenatal testing study at age 42. Her echocardiogram and MRA were normal. The proband's daughter (5-III-1, 18 y) carries the *BGN* variant. She has had a normal echocardiography, joint hypermobility but no dislocations or contractures, very petite facial features and easy bruising possibly relating to dyspraxia. The proband reported that her mother (5-I-1, 72 y) is an asymptomatic carrier for the *BGN* variant. The proband's mother was a high-level competitive handball player and declines comprehensive vascular imaging.

Family 6 – The male proband (6-III-1, 36 y) had a sinus of Valsalva that measured 34 mm on echocardiography and 29x27x26 mm on MRA (z-score of 0.61). He received a clinical diagnosis of Beals syndrome in childhood. As a baby, he was noted to have a unilateral crumpled ear and severe bilateral talipes requiring multiple casting and surgeries. He was tall (192 cm) with significant camptodactyly of the fingers and toes. Skeletal X-rays revealed laevoconvex scoliosis, end plate fracture at L1, a fusion of the fifth lumbar bone with pelvis and mild form of spina bifida occulta, laterolisthesis of L3 on L4, reduced disc space L4/L5 with retrolisthesis, facet arthrosis of lower lumbar spine, decreased bone density. He also had flat feet and joint contractures, self-reported teeth overcrowding. There was no vision or hearing problems. The mother of the proband (6-II-2, 69 y) presented with hypertension, hypercholesterolaemia, mild joint problems, osteopenia, congenital hammer toes, pes planus, and recurrent locking of finger joints. Her X-rays revealed small T12 ribs, degenerative spondylolisthesis (most pronounced at L5/S1), facet arthrosis, sclerotic sacroiliac joints, calcification of hip joint capsule and pelvic ligaments, as well as small osteophytes and Baastrup syndrome at several levels of the spine. She had a normal stature (170 cm). The maternal aunt (6-II-3, 71 y) had hypertension and a tricuspid aortic valve, but normal echocardiography, and no aneurysms on MRA. Radiography of her spine revealed moderate degenerative changes of C4-7 with normal alignment. She had flat feet, hypermobility of little fingers (Beighton score 2 out of 9), malar hypoplasia and normal stature (170 cm). She self-reported that she had weak ankles as a child, and backpain following an accident but denied prior dislocations or hernia. She had a Ghent assessment, scoring only for myopia. Other relevant negatives on examination include no hypertelorism, milia, dystrophic scars or piezogenic papules, as well as normal skin extensibility and uvula. This aunt's daughter (6-III-4, 50 y) showed a normal aortic root size and no evidence of aneurysm formation. Imaging of brain and neck vessels revealed bilaterally absent posterior communicating arteries and short proximal left common carotid artery stenosis. She had hypertension, self-reported backpain since the age of 10, dolichocephaly, malar hypoplasia, and myopia (-3.75/-4 dioptres). She presented with long legs but normal stature (167 cm) and joint hypermobility in knees and hips. Segregation analysis confirmed the presence of the *BGN* variant in these three female family members.

Family 7 – The male proband (7-II-3, 11 y) presented with hypermobility in both thumbs, left elbow and right knee (Beighton score of 4 out of 9), and reported joint pain. He also had a short stature (131 cm), bilateral planovalgus deformity, a highly arched palate, delayed wound healing and learning problems. Echocardiography showed normal aorta and cardiac function. He also carried a heterozygous *de novo* VUS in *RORA* (NM_134261.2): c.1283T>C (p.Leu428Pro). The *BGN* variant was also identified in the unaffected mother (7-I-1, 43 y). Her computer tomography (CT) of the aorta was normal, and her clinical exam did not show signs of a connective tissue disorder (Beighton 0 out of 9). She had hypertension, migraine, and a normal stature (164 cm). No family history of aortic aneurysm or dissection was reported.

Family 8 – The male proband (8-II-2, 72 y) had aneurysms in both common femoral arteries (36 mm and 27 mm), right popliteal artery (16 mm), left common iliac artery (32 mm), and a mild dilation of the upper descending thoracic aorta (37 mm). He also had hypertension, mitral valve prolapse, peripheral arterial disease and coronary artery disease. He had joint hypermobility in the metacarpophalangeal joints when he was younger, now he had a Beighton score of 0 out of 9. He had mild skin hyperextensibility, flat feet, multiple fractures, and mild scoliosis. The latter might be related to two vertebral fractures due to accidents. He had downslanting eyes, malar hypoplasia, myopia (-2/-1.25 dioptres) and astigmatism. He had a normal stature (180 cm). His brother (8-II-1, deceased), an obligate carrier of the *BGN* variant, drowned when he was 30 years old. The brother's two daughters both tested positive for the *BGN* variant. The

first daughter (8-III-1, 37 y) had flat feet, joint hypermobility (left thumb apposition to forearm and palms to floor), mild skin hyperextensibility, striae and easy bruising. She presented with a positive Walker-Murdoch sign, but a negative thumb sign. She had a Beighton score of 2 out of 9, and a normal stature (166 cm). The CT of the aorta and ultrasound of the heart were normal. The other daughter (8-III-2, 35 y) had congenital pectus excavatum, low-set posteriorly rotated ears, mild myopia (-1.25/-1.25 dioptres), astigmatism, flat feet, joint hypermobility (bilateral thumb apposition to forearm and palms to floor), and mild skin hyperextensibility. She presented with a positive Walker-Murdoch sign, but a negative thumb sign. She had a Beighton score of 3 out of 9, and a normal stature (171 cm). The CT of the aorta and ultrasound of the heart were normal. There was no family history of aneurysms.

Family 9 – The male proband (9-II-3, 55 y) had a dissection in his left vertebral artery, and imaging revealed dilated right vertebral artery, renal arteries, and basilar trunk. He had multiple fractures and osteoporosis. He also carried a relatively common VUS in *LRP5* (NM_002335.4): c.3107G>A (p.Arg1036Gln). The brother of the proband (9-II-4, 53 y) also carried the *BGN* variant and had osteoporosis and a bleeding disorder. They had normal echocardiograms at the age of 48 and 49, respectively. Their mother (9-I-5, deceased), an obligate carrier of the *BGN* variant, had a bleeding disorder and died from hepatic cancer when she was 76 years old. One daughter of the proband (9-III-1, deceased) died from an acute respiratory distress syndrome in the context of pneumonia and diabetes type 1 at the age of 2. The three children of the proband's sister (9-II-5) are asymptomatic (9-III-4, 9-III-5, and 9-III-6). There is no family history of arterial aneurysm on the maternal side of the family.

Family 10 – The male proband (10-II-2, 59 y) presented at age 51 with ascending aorta aneurysm (40 mm, z-score of 2.56) and severe saccular aortic root aneurysm (80 mm, z-score of 9.51) from the left aortic sinus. He underwent minimal invasive Bentall surgery a few weeks following diagnosis. Other cardiovascular features include renal artery dissection, cardiomyopathy bicuspid aortic valve with mild insufficiency, calcifications, stenosis, and an aneurysm of the left coronary sinus. He had pronounced pectus excavatum, osteoporosis, and recurrent pneumothorax, but no other skeletal manifestations. He also had myopia, easy bruising and hypercholesteremia. The proband presented with a normal stature (184 cm). The proband is also carrier of a *SLC2A10* variant. His brother (10-II-1, 63 y) also carried the *BGN* variant and had an aortic root diameter of 36 mm (z-score of 0.47) and ascending aorta of 35 mm (z-score of 1.07). He had a short stature (168 cm). The sister of the proband (10-II-3, 51 y) also carried this *BGN* variant and had an aortic root diameter of 36 mm, which corresponds with an aortic z-score of 2.11. She had an aneurysm in the arteria cerebri media, osteopenia, and retinitis pigmentosa with complete blindness. She presented with a short stature (163 cm). The mother of these siblings (10-I-1) is an obligate carrier of the *BGN* variant. She died at the age of 58 due to lung cancer. She had hypertension, a tall stature (180 cm), myopia and diabetes/obesity/hypercholesterolemia/ arteriosclerotic cardiovascular disease for which she received percutaneous coronary intervention.

Family 11 – The male proband (11-II-1, 45 y) had a diagnosis of trichorhinophalangeal syndrome (TRPS), which was confirmed by a likely pathogenic truncating variant in *TRPS1* (NM_014112.4): c.3252C>A (p.Tyr1084*). He also had an aortic root dilation of 45 mm (z-score of 3.43), asymptomatic arterial ectasia/aneurysms of coeliac, mesenteric, hepatic, femoral, popliteal, tibioperoneal arteries for which stenting, and bypass procedures were performed. Magnetic resonance imaging of the brain showed evidence of chronic ischemia, but normal vessels. The heart was structurally normal. He had relative macrocephaly, keratoconus, proptosis, mild ptosis, a round face and a pointed nasal tip. He also presented with hypermobility, short stature (160 cm), brachydactyly, and bilateral avascular necrosis of the hip, for which he received hip replacement. The latter four features could also be the consequence of TRPS.

Family 12 – The male proband (12-II-1, deceased) died aged 29 years. He had had an aortic dissection and emergency surgery aged 27 years, with aortic valve and ascending aorta replacement. Then, he had a further dissection of the descending aorta aged 28 years. He was known to have a ventricular septal defect. The proband had a marfanoid appearance; he had tall stature (196 cm), was thin, had scoliosis and pectus excavatum. He had dolichostenomelia, arachnodactyly, pes planus, contractures of his fingers and toes. He suffered with joint dislocations and hypermobility. He had a highly arched palate, dental overcrowding, several dental extractions, and myopia. He had suffered from

seizures from an early age, which ceased at age 18 years. He wore callipers on his legs as a child to aid walking and suffered from muscle spasms and cramps. He had developmental delay, having first walked at the age of 2 years. He was thought to have some additional learning needs and did not obtain any educational qualifications. He had mental health issues. The proband inherited the *BGN* variant from his mother (12-I-2, 59 y), who had reduced elbow extension, mild brachydactyly, spatulous fingers, down-slanting palpebral fissures, and diabetes type II. She had a normal stature (170 cm) and wore glasses for myopia. She had no evidence of scoliosis or pectus. She was hypermobile when younger. The proband's half-sister (12-II-3, 22 y) was tall (177 cm) and had dolichostenomelia, hypermobility, stretch marks on her underarms and legs, reduced elbow extension, joint pain, high-arched palate, myopia, and easy bruising. Her echocardiogram was normal. She had a diagnosis of postural orthostatic tachycardia and polycystic ovary syndrome. She had dyslexia but otherwise normal learning.

Family 13 – The female proband (13-III-2, 59 y) presented with mild aortic dilatation of the aortic root (38 mm, z-score of 1.7) and ascending aorta (41 mm, z-score of 2.81). She had a common origin of the brachiocephalic artery and left common carotid artery. Otherwise, her whole body MRA was normal. She had hypertension, pectus deformity, normal stature (158 cm), proptosis, and dermatomyositis. Her mother (13-II-2, deceased), an obligate carrier of the *BGN* variant, had kyphosis and osteoporosis and died from complications (presumably a dissection) in a 'mega-aorta' at the age of 73. Both daughters of the proband (13-IV-2, 37 y and 13-IV-7, 30 y) carry the *BGN* variant, but had a normal echocardiogram, abdominal ultrasound, and whole body MRA. They also had normal statures (163 cm and 160 cm, respectively). One of these daughters presented with muscle weakness in her shoulder girdle muscles. The proband's son (13-IV-4, 35 y) is a hemizygous carrier of the *BGN* variant and showed a mild dilatation of the sinotubular junction (36 mm) and aortic root (35 mm, z-score of 0.72), but his whole body MRA was otherwise normal. He also had a unilateral inguinal hernia, normal stature (180 cm) and mild learning problems. The *BGN* variant was also found in a grandson of the proband (13-V-8, 4 y), but he was non-dysmorphic and had a normal aorta. In addition, the *BGN* variant was present in the following granddaughters of the proband: 13-V-2 (1 y), 13-V-6 (1 y), 13-V-7 (2 y), and 13-V-13 (12 y). They were either not yet clinically assessed (13-V-2, 13-V-6) or had a normal echocardiogram (13-V-7, 13-V-8, 13-V-13).

Supplementary Figure 2

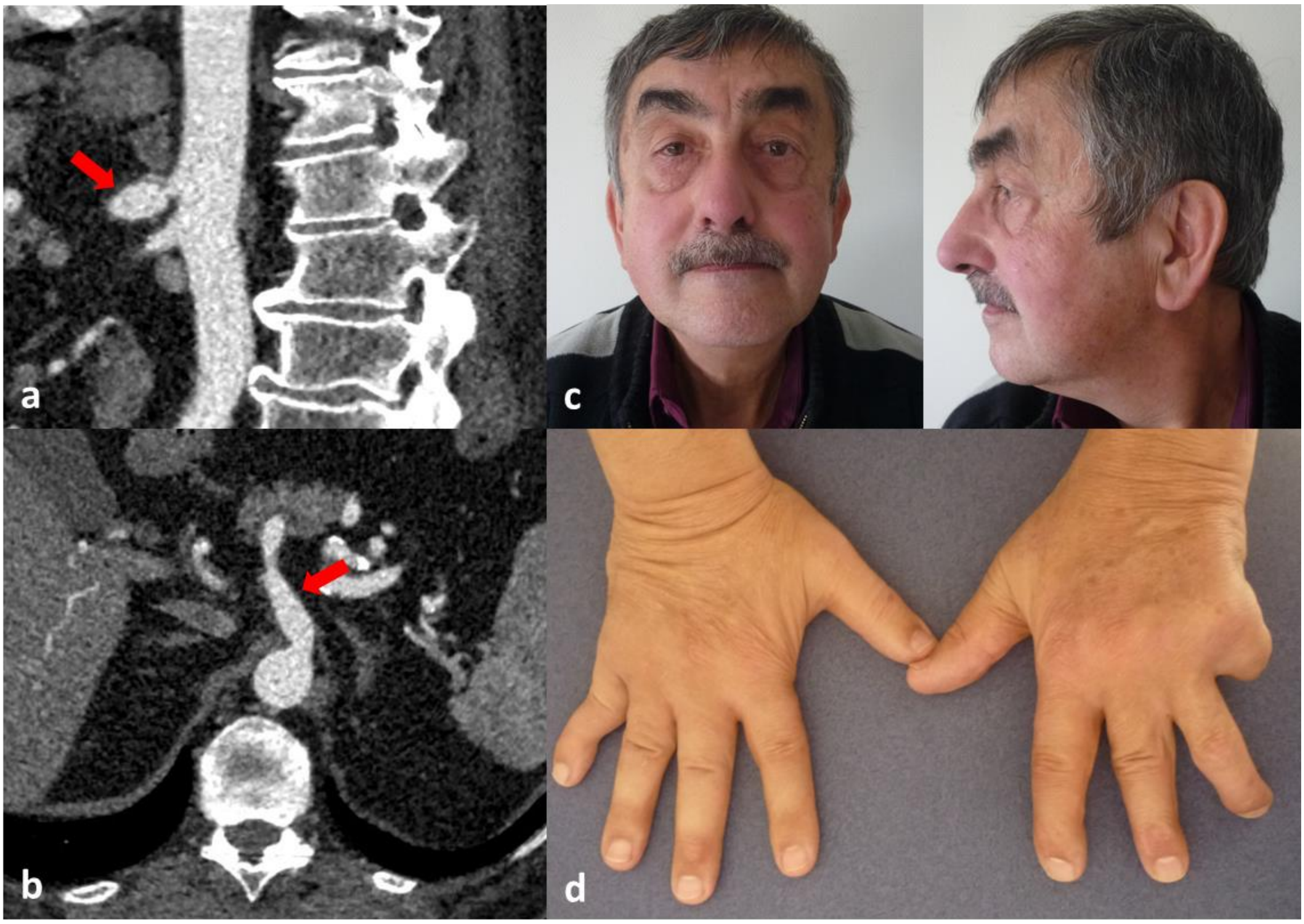

Supplementary Figure 2. Phenotypical characteristics of the proband of family 3 (3-I-2). Panel a and b: computerized tomography scan with abdominal sagittal and transversal section showing a coeliac aneurysm (red arrows). Panel c: craniofacial dysmorphism with hypertelorism, malar flattening. Panel d: brachydactyly, and camptodactyly of left fifth finger secondary to a Dupuytren's disease. Written informed consent was obtained for publication of these photographs.

Supplementary Methods

Clinical checklist

Referring Center:____________________ Contact person:_____________________
Your local patient ID: _______________ DOB:____________

Gene Mutation and family data

BGN1 mutation c.________________ leading to p.________________
De novo mutation *Y/N/unknown*
Family history *Y/N/unknown* Segregation in family tested *Y/N/unknown*
If this is checklist of a family member: relationship to proband:_________________________
If deceased: age at death:_____yrs; cause of death:__________________________________

Age at assessment: _______yrs *Height:_____cm* *Weight:____kg*
Sex: Male/Female

Craniofacial features:

Hypertelorism *Y/N/unknown*		Blue sclerae	*Y/N/unknown*
Downslant palpebral fissures *Y/N/unknown*		Malar hypoplasia	*Y/N/unknown*
Retrognathia *Y/N/unknown*		Dolichocephaly	*Y/N/unknown*
High arched palate *Y/N/unknown*		Frontal bossing	*Y/N/unknown*
Gingival hypertrophy *Y/N/unknown*			
Ectopia lentis	*Y/N/unknown*	Retinal detachment	*Y/N/unknown*
Cataract	*Y/N/unknown*	Glaucoma	*Y/N/unknown*
Exotropia	*Y/N/unknown, if yes R/L*	Proptosis	*Y/N/unknown*
Myopia	*Y/N/unknown, if yes diopters L____/R______*		
Cleft palate	*Y/N/unknown*	Broad/bifid uvula	*Y/N/unknown*

Craniosynostosis *Y/N/unknown, if yes which sutures*__________________________

Skeletal features:

Dolichostenomelia	*Y/N/unknown*	Short stature	*Y/N/unknown*
Arachnodactyly	*Y/N/unknown*	Brachydactyly	*Y/N/unknown*
Joint hyperlaxity	*Y/N/unknown*	Camptodactyly	*Y/N/unknown*
Spatulous fingers	*Y/N/unknown*		
Club foot	*Y/N/unknown*	Spondylolisthesis	*Y/N/unknown*
Flat feet	*Y/N/unknown*	Osteo-arthritis	*Y/N/unknown*
Osteoporosis	*Y/N/unknown*	Fractures	*Y/N/unknown, #:________*

Pectus deformity *Y/N/unknown, if yes carinat/excavatum/asymmetry, surgery at age____yrs*
Cervical spine instability *Y/N/unknown, if yes surgery at age______yrs*
Scoliosis *Y/N/unknown, if yes surgery at age______yrs*
Joint dislocation *Y/N/unknown, if yes which joints*_______________________
Joint contractures *Y/N/unknown, if yes which joints*_______________________

Skin/integmentum features:

Thin, translucent skin	*Y/N/unknown*	Easy bruising	*Y/N/unknown*
Striae	*Y/N/unknown*	Atrophic scarring	*Y/N/unknown*
Delayed wound healing	*Y/N/unknown*	Hypertrichosis	*Y/N/unknown*

Hernia *Y/N/unknown*, if yes inguinal/umbilical/hiatal, other:__________________
Dural ectasia *Y/N/unknown*

Clinical checklist

Cardiovascular features:
Aortic dissection/rupture: *Y/N/unknown, if yes: age_____yrs, size before dissection:____mm; localization of dissection/rupture:____*
Aortic surgery: *Y/N/unknown, if yes: elective yes/no; age_____yrs, size of aorta at time of surgery:____mm; localization of aortic surgery:____________________________________*

Aortic root aneursym:	*Y/N/unknown, if yes size _____mm (Z-score=______)*
Ascending aortic aneuryms	*Y/N/unknown, if yes size _____mm (Z-score=______)*
Other aortic aneurysm	*Y/N/unknown, if yes localization:_____________________*
Arterial aneurysm	*Y/N/unknown, if yes localization:_____________________*
Aortic tortuosity	*Y/N/unknown*
Arterial tortuosity	*Y/N/unknown, if yes localization:____________________*
Mitral valve prolapse	*Y/N/unknown* Patent ductus arteriosus *Y/N/unknown*
Bicuspid aortic valve	*Y/N/unknown* Atrial septal defect *Y/N/unknown*

Please list all cardiovascular medications (past and present)

______________ Age started________ Age stopped________Still Taking (Y/N/unknown)
______________ Age started________ Age stopped________Still Taking (Y/N/unknown)
______________ Age started________ Age stopped________Still Taking (Y/N/unknown)

Other features:

Pneumothorax	Y/N/unknown
Tooth enamel defect	*Y/N/unknown*
Eosinophilic esophagitis	*Y/N/unknown*
Inflammatory bowel disease	*Y/N/unknown*
Food allergy	*Y/N/unknown*
Severe allergy	*Y/N/unknown*

Neuromuscular features:

Myopathy	*Y/N/unknown*
Mild learning problems	*Y/N/unknown*
Relative macrocephaly	*Y/N/unknown*
Dilated ventricles	*Y/N/unknown*

Other features:

____

____

Clinical checklist

Pregnancy:

If female, number of pregnancies ______

Duration of each pregnancy (weeks) _______, _______, _______, _______, _______

Did vascular dissection or rupture occur with any pregnancy (Y/N/unknown)

If yes, which pregnancy 1st, 2nd, 3rd, 4th, 5th, other ______

If yes, which blood vessel(s) _______________________________

If yes, at what gestational age _______, or # of days post delivery _______

If yes, what was the dimension of each affected blood vessel:

_______, _______, _______, _______

Which pregnancies were associated with breast feeding/pumping

1st, 2nd, 3rd, 4th, 5th, other ______

Did death occur as a result of a pregnancy-associated vascular event (Y/N/unknown)

Did uterine rupture occur with any pregnancy (Y/N/unknown)

If yes, which pregnancy 1st, 2nd, 3rd, 4th, 5th, other ______

If yes, at what gestational age _______, or at delivery (Y/N/unknown)

Did death occur as a result of uterine rupture (Y/N/unknown)

Were there other pregnancy-associated complications (Y/N/unknown)

If yes, please explain ____

____

Supplementary Figure 3

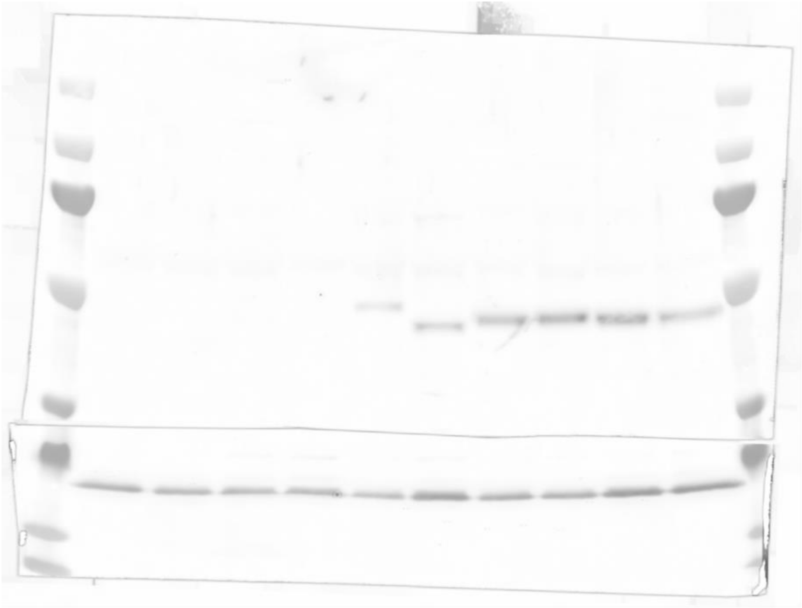

Supplementary Figure 3. Uncropped image of the Western Blot shown in Figure 2.

Supplementary Figure 4

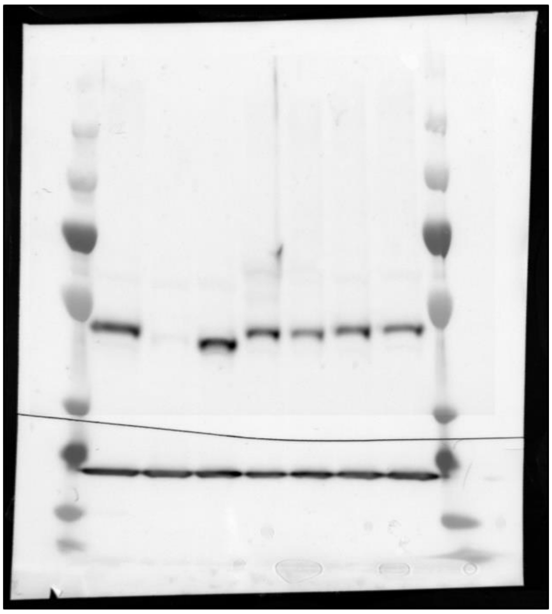

Supplementary Figure 4. Uncropped image of the Western Blot shown in Supplementary Figure 1.
